# Niche-specific gene expression in a parasitic nematode; increased expression of immunomodulators in *Teladorsagia circumcincta* larvae derived from host mucosa

**DOI:** 10.1038/s41598-017-07092-0

**Published:** 2017-08-03

**Authors:** Tom N. McNeilly, David Frew, Stewart T. G. Burgess, Harry Wright, David J. Bartley, Yvonne Bartley, Alasdair J. Nisbet

**Affiliations:** Moredun Research Institute, Edinburgh, United Kingdom

## Abstract

Metazoan parasites have to survive in many different niches in order to complete their life-cycles. In the absence of reliable methods to manipulate parasite genomes and/or proteomes, identification of the molecules critical for parasite survival within these niches has largely depended on comparative transcriptomic and proteomic analyses of different developmental stages of the parasite; however, changes may reflect differences associated with transition between developmental stages rather than specific adaptations to a particular niche. In this study, we compared the transcriptome of two fourth-stage larval populations of the nematode parasite, *Teladorsagia circumcincta*, which were of the same developmental stage but differed in their location within the abomasum, being either mucosal-dwelling (MD) or lumen-dwelling (LD). Using RNAseq, we identified 57 transcripts which were significantly differentially expressed between MD and LD larvae. Of these transcripts, the majority (54/57) were up-regulated in MD larvae, one of which encoded for an ShKT-domain containing protein, Tck6, capable of modulating ovine T cell cytokine responses. Other differentially expressed transcripts included homologues of ASP-like proteins, proteases, or excretory-secretory proteins of unknown function. Our study demonstrates the utility of niche- rather than stage-specific analysis of parasite transcriptomes to identify parasite molecules of potential importance for survival within the host.

## Introduction

Many species of parasites have evolved complex and intricate lifecycles involving intimate associations with their intermediate, definitive, paratenic and, in some cases, vector hosts. Even within directly-transmitted species, such as the Strongylid nematodes which infect ruminants and other mammalian hosts, successive developmental stages are subject to a plethora of different environmental pressures and stimuli and their adaptations to these environmental factors are reflected in the diversity of their transcriptomic responses at each life stage throughout development (e.g. refs 1–4). In these nematodes, the transition to a parasitic existence, when the infective larval stage is consumed by the definitive host, represents a sea-change in which a free-living, non-feeding organism exposed to fluctuating temperatures and weather conditions becomes a parasitic organism living in darkness, feeding on protein, growing rapidly and, crucially, negotiating and subverting the host’s immune responses^5^. In the economically-important strongylid nematode *Teladorsagia circumcincta*, this transition from the infective third larval stage (L3) on pasture to the fourth larval stage (L4) which resides in the abomasum (true stomach) of sheep, is associated with the upregulation of suites of genes involved in nutrition, growth, host immunomodulation and respiration in a micro-aerobic environment^6^.

Although the paradigm for the initial parasitic development of *T. circumcincta* is often simplified to describe the L3 entering the gastric gland, moulting to L4 and emerging into the abomasal lumen as an immature adult, early descriptions of the lifecycle describe three populations of L4 worms in the abomasum^7, 8^: Thus, after entry of the iL3 into the gastric pits and glands and moulting to L4, the larvae either, (i) fail to develop further; (ii) emerge into the lumen immediately after moulting or, (iii) remain in the mucosa for a longer period, emerging into the lumen as late L4 or early adults^7^. The dynamics and biology of the latter two populations is of great interest from a biological, parasitological and immunological point of view as these worms represent niche-specific variants of the same age and stage of the species^9^. As a consequence, differences with the trancriptome and proteome between these two populations are more likely to represent transient adaptations to mucosal or luminal environments, rather than reflecting the more substantial differences associated with transition between individual developmental stages of the parasite lifecycle. One particularly intriguing aspect of the discrimination between populations of *T. circumcincta* L4 which inhabit the abomasal lumen or are intimately associated with the abomasal mucosa is their different requirements to immunomodulate the host dependent on niche, and this may be highly relevant to the development of novel parasite control strategies^10^. Indeed, immunomodulation by *T. circumcincta* L4 is likely to be critical for survival of the parasite within the host as immune responses directed against this stage of the parasite are highly correlated with protection^11, 12^.

The aim of the work presented here is therefore to discover which genes are differentially transcribed between the populations of worms which are in intimate contact with the host abomasal mucosa and those worms which exist in the less immunologically hostile environment of the abomasal lumen.

## Results

### Gross morphology of lumen dwelling and mucosal dwelling *T. circumcincta* L4

**Figure 1:**
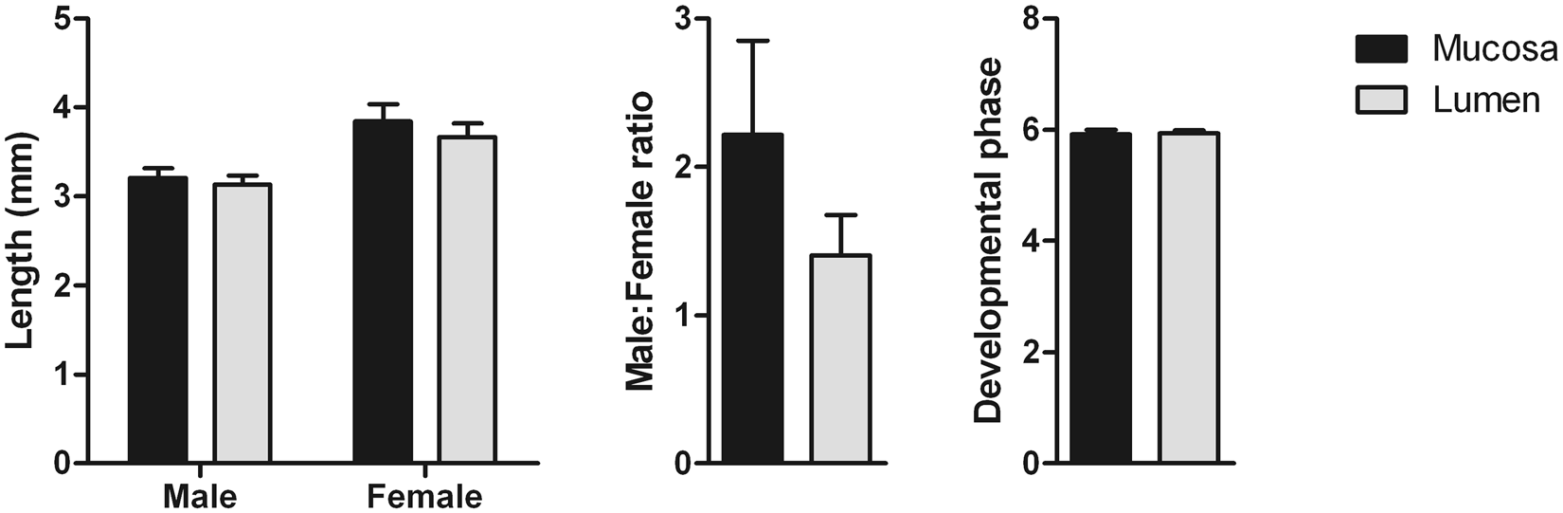
Gross morphological characteristics of MD and LD *T. circumcincta* L4. Six helminth-free lambs were infected with *T. circumcincta* L3 and seven days later fourth-stage larvae (L4) were harvested from either the abomasal mucosa (Mucosa) or abomasal lumen (Lumen). Lengths, male:female ratios and developmental phases were recorded for 50 mucosal and 50 lumen-dwelling larvae from each individual lamb. Development phases were determined according to ref. 8, with phases 4 and 8 representing early and late stage L4, respectively. No significant differences in any gross parasitological parameters were identified.

Gross morphological data is shown in Fig. 1. There was no significant difference in worm lengths, sex (including no site × sex interaction), or stage of development between luminal dwelling (LD) and mucosal dwelling (MD) larvae. The mean stage of development was phase 6 for both LD and MD larvae^8^, indicating that both populations were mid-L4 stage.

### Assembly and analysis of the *T. circumcincta* transcriptome

Assembly of the collated *T. circumcincta* sequencing data resulted in the generation of 28,155 contigs/isotigs and 67,445 singletons. The N50 value of the contigs >100 bp was 731 bp, maximum contig length 6481 bp and the number of contigs >1 kb was 4340. Following filtering for contig sequences of <100 bp, 28,143 contig sequences were retained for downstream analyses. Completeness of the *T. circumcincta* transcriptome assembly was assessed via Benchmarking Universal Single-Copy Orthologs (BUSCO) analysis^13^ using the Nematoda lineage dataset as a reference. This was also compared to the current transcript predictions for the *T. circumcincta* genome available within Wormbase-Parasite (http://parasite.wormbase.org/Teladorsagia_circumcincta_prjna72569/Info/Index/). The analysis demonstrated that, as observed with other nematode transcriptomes the completeness level as assessed by BUSCO was relatively low (23% for our *T. circumcincta* assembly and 38% for the Wormbase-Parasite *T. circumcincta* predicted transcripts (BioProject: PRJNA72569)) however the level of completeness for each transcriptome was comparable. Of the 28,143 retained contig sequences, BLASTx analysis identified 17,863 (63.4%) with significant (<1e^−02^) homology to sequences deposited in the NCBI nr database (25^th^ July 2014 release). The remaining 10,280 contigs had either no or low homology (E-value >1e^−02^) to sequences within the NCBI nr database, potentially representing *T. circumcincta* transcripts of, as yet, undefined function.

### Determination of differentially expressed transcripts between lumen dwelling and mucosal dwelling larvae

**Figure 2:**
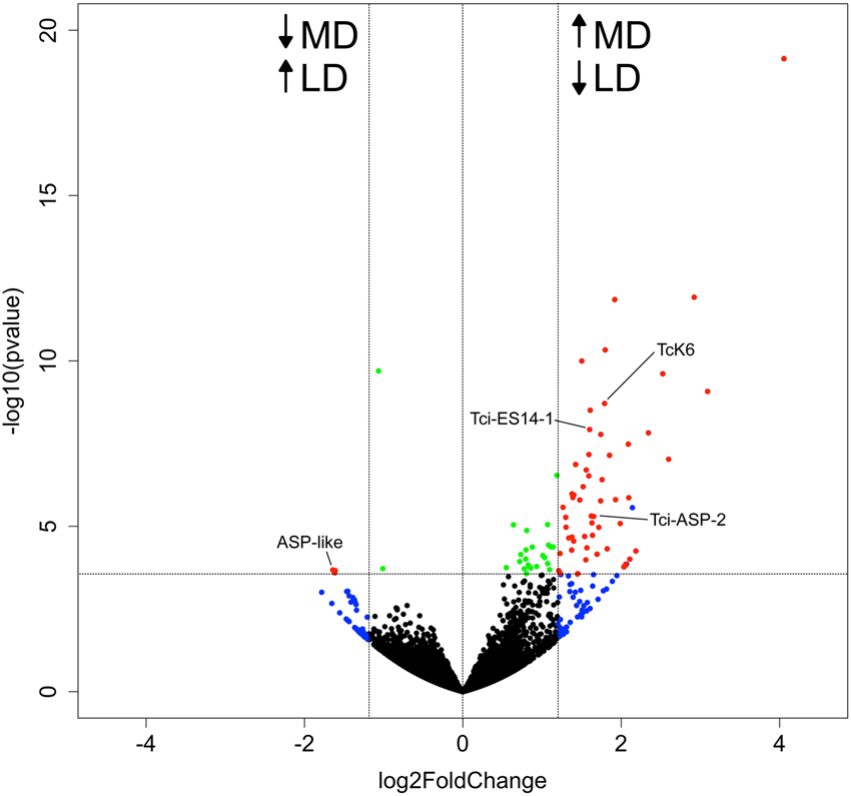
Volcano plot showing log2 (fold change, FC) against −log10 (p-value) of transcripts identified by RNASeq analysis of MD and LD *T. circumcincta* L4. Transcripts significantly differentially expressed between three replicates of luminal and mucosal dwelling *T. circumcincta* larvae are highlighted in red (FDR corrected p-value of ≤(FDR corrected p-v) and a FC ≥± 1.5). Green dots indicate significant transcripts with a FC ≤ 1.5; blue dots indicate transcripts with a FC ≥± 1.5 but with an FDR corrected p-value of ≥0.05; whilst black dots indicate non-significant transcripts with a FC ≤ 1.5. Arrows further indicate the direction of up-regulation and down-regulation of transcripts in mucosal dwelling (MD) or lumen dwelling (LD) larvae.

The total number of Hi-Seq reads for each of the six RNA sample sets mapping to each contig/isotig of the *T. circumcincta* transcriptome is described in Table 1. RNA-Seq obtained approximately 28 to 34 million raw sequence reads for each of the six libraries (three each from luminal and mucosal isolated larvae). Trimmed reads were then aligned to the *T. circumcincta* transcriptome using the CLC Genomics Workbench (Version 8, Qiagen Ltd) and normalised read count tables generated for assessment of differential gene expression. Differential expression analysis using DESeq. 2 (Version 1.8.1) based on a fold change cut-off ≥±1.5 between LD and MD larvae samples and an FDR corrected *p-*value of ≤0.05, showed that 57 transcripts were significantly differentially expressed between LD and MD larvae. Of these differentially expressed transcripts, 54 were up-regulated and three were down-regulated in MD larvae, and Fig. 2 shows a volcano plot of log_2_ (fold change) against −log_10_ (p-value) for the entire *T. circumcincta* dataset, highlighting the bias towards increased gene expression within the MD larvae samples.

**Table 1:** Total number of Illumina Solexa Hi-Seq reads for each of the six RNA sample sets mapping to each contig/isotig of the *T. circumcincta* transcriptome.

Sample ID	Sheep ID	Total reads	Aligned reads	% aligned reads	Contigs/isotigs with aligned reads
LD 1	1	34,915,923	26,488,590	75.8	21,863
LD 2	2	28,710,123	21,826,128	76.0	21,721
LD 3	3	32,018,622	24,318,979	75.9	21,173
MD 1	1	30,078,634	22,815,445	75.8	21,142
MD 2	2	27,789,924	21,042,440	75.7	21,209
MD 3	3	32,574,992	24,706,807	75.8	21,169

LD = lumen-dwelling larvae; MD = mucosal dwelling larvae.

Of the differentially expressed transcripts, 23/54 of the MD up-regulated and 3/3 MD down-regulated transcripts had significant BLASTx hits (Table 2). In addition, 43 of the differentially expressed transcripts were homologues of genes present within the *T. circumcincta* genome assembly, PRJNA7256 (Table 2 and Supplementary Table S1). Up-regulated transcripts in MD larvae included five isotigs with homology to venom allergen/ASP-like proteins, five isotigs with homology to parasite proteases, one isotig (20922) with homology to a *Stichodactyla helianthus* toxin (ShKT)-related protein from the salmon louse *Lepeophtheirus salmonis*, and 12 isotigs with homology to parasite proteins of unknown function, of which nine were homologues of proteins previously identified in ruminant nematode excretory-secretory (ES) products: five were homologues of a putative L3 ES protein from *Ostertagia ostertagi* (accession no. CAH23216.1) and four were homologues of a previously identified 15 kDa excretory/secretory protein of *Haemonchus contortus* (accession no. CDJ91363.1). Thirty-four (60.0%) of the differentially expressed isotigs possessed a signal peptide sequence.

**Table 2:** Details of isotigs differentially expressed in mucosal vs. luminal *T. circumcincta* L4, with FDR adjusted p-value ≤ 0.05.

Isotig accession no.	Closest homologue in database (nematode species: NCBI accession no.)	BLAST eValue	% homology	Fold change	Tci-gene ID^e^
*Venom allergen/ASP-like* (*VAL*) homologues					
isotig04636	activation associated secreted protein (*O.ostertagi*:CAN84557.1)	2E-4	35	5.49	TELCIR_02385
isotig05835	SCP extracellular domain containing protein (*H. contortus*: CDJ88115.1)^**a**^	5.5E-9	45.9	3.03	TELCIR_20852
isotig03532	SCP extracellular domain containing protein (*H. contortus*: CDJ88115.1)	1.6E-11	54.4	2.66	TELCIR_20852
isotig05834	SCP extracellular domain containing protein (*H. contortus*: CDJ88117.1)	2.3E-13	63	2.53	TELCIR_20852
isotig23217	SCP extracellular domain containing protein (*H. contortus*: CDJ88117.1)*	7.6E-6	58.7	2.31	TELCIR_11944
isotig22376	ancyclostoma-secreted protein-like protein (*O. ostertagi*: CAD56659.1)	5.5E-46	59.8	−2.60	TELCIR_04994
isotig00647	ancyclostoma-secreted protein-like protein (*O. ostertagi*: CAD56659.1)	1.1E-46	62.5	−2.62	TELCIR_17222
isotig00650	ancyclostoma-secreted protein-like protein (*O ostertagi*: CAD56659.1)	2.3E-7	84.8	−2.70	TELCIR_17222
*Proteases*					
isotig12346	Peptidase M12A domain containing protein (*H. contortus*: CDJ93881.1)	4.2E-15	46.6	4.39	TELCIR_06002
isotig22769	Metalloprotease IV, partial (*Ostertagia ostertagi*: AAS47831.1)	6.6E-4	66.6	2.87	TELCIR_06002
isotig21073	blastula protease 10, partial (*Clonorchis sinensis*: GAA56304.1)	1.67E-5	53	8.53	TELCIR_17994
isotig21358	blastula protease 10, partial (*Clonorchis sinensis*: GAA56304.1)*	1.34E-4	55	6.75	TELCIR_17994
isotig20101	blastula protease 10, partial (*Clonorchis sinensis*: GAA56304.1)	9.22E-9	56	3.03	TELCIR_17994
*Shk-domain* ^*d*^ *containing protein*					
isotig20922	protein ZK643.6 (*Lepeophtheirus salmonis*: ADD24043.1)^**b**,*^	2.7E-3	58	3.20	TELCIR_17916
*Parasite proteins of unknown function*					
isotig15866	putative L3 ES protein (*O. ostertagi*:CAH23216.1)*	1.7E-3	44	2.59	TELCIR_14600
isotig15536	putative L3 ES protein (*O. ostertagi*:CAH23216.1)^**c**,^*	4.1E-5	46	2.56	TELCIR_14600
isotig17294	putative L3 ES protein (*O. ostertagi*:CAH23216.1)*	1.5E-6	51	2.43	TELCIR_02611
isotig16815	putative L3 ES protein (*O. ostertagi*:CAH23216.1)*	7.4E-6	50	2.18	TELCIR_04250
isotig17499	putative L3 ES protein (*O. ostertagi*:CAH23216.1)*	1.2E-3	50	2.03	TELCIR_00024
isotig02797	15 kDa excretory/secretory protein (*H. contortus*: CDJ91363.1)	3.5E-16	57.2	3.43	TELCIR_00013
isotig02796	15 kDa excretory/secretory protein (*H. contortus*: CDJ91363.1)*	2.5E-14	52	3.09	TELCIR_00013
isotig02794	15 kDa excretory/secretory protein (*H. contortus*: CDJ91363.1)*	2.6E-14	51.7	2.73	TELCIR_00013
isotig02795	15 kDa excretory/secretory protein (*H. contortus*: CDJ91363.1)	3.5E-16	56.9	2.46	TELCIR_00013
isotig06110	hypothetical protein Y032_0222g2621 (*Ancylostoma ceylanicum*: EYB90231.1)	2.8E-3	43	2.41	TELCIR_04716
isotig11270	hypothetical protein Y032_0101g3382 (*Ancylostoma ceylanicum*: EYC02233.1)*	1.8E-5	43.7	1.96	TELCIR_20437
isotig13663	hypothetical protein PC101070.00.0 (*Plasmodium chabaudi chabaudi*: CAH88354.1*)*	6.5E-7	73.3	16.44	No hits found

*Denotes presence of a signal peptide. ^a^Isotig represents Tci-ASP-2; ^b^isotig represents TcK6; ^c^isotig represents Tci-ES14-1. *O. ostertagi* = *Ostertagia ostertagi*; *H. contortus* = *Haemonchus contortus*. ^d^ *Stichodactyla helianthus* toxin-domain. ^e^Identity of homologous gene from *Teladorsagia circumcincta* genome assembly (PRJNA72569): http://parasite.wormbase.org/Teladorsagia_circumcincta_prjna72569.

Relationships amongst those differentially-expressed molecules which had significant homologies to other molecules in public databases were inferred by alignment of the nucleotide sequences using ClustalX version 2.0.10, employing Neighbour-joining analysis and the resulting relationship tree constructed using TreeView is shown in Supplementary Fig. S1. This shows distinct groupings of related differentially-expressed, functionally related genes and that differentially regulated venom allergen/ASP-like transcripts segregate into four distinct clusters. Details of transcripts with no known homology are shown in Supplementary Table S1.

### Stage-specific expression of transcripts upregulated in MD *T. circumcincta* L4

**Figure 3:**
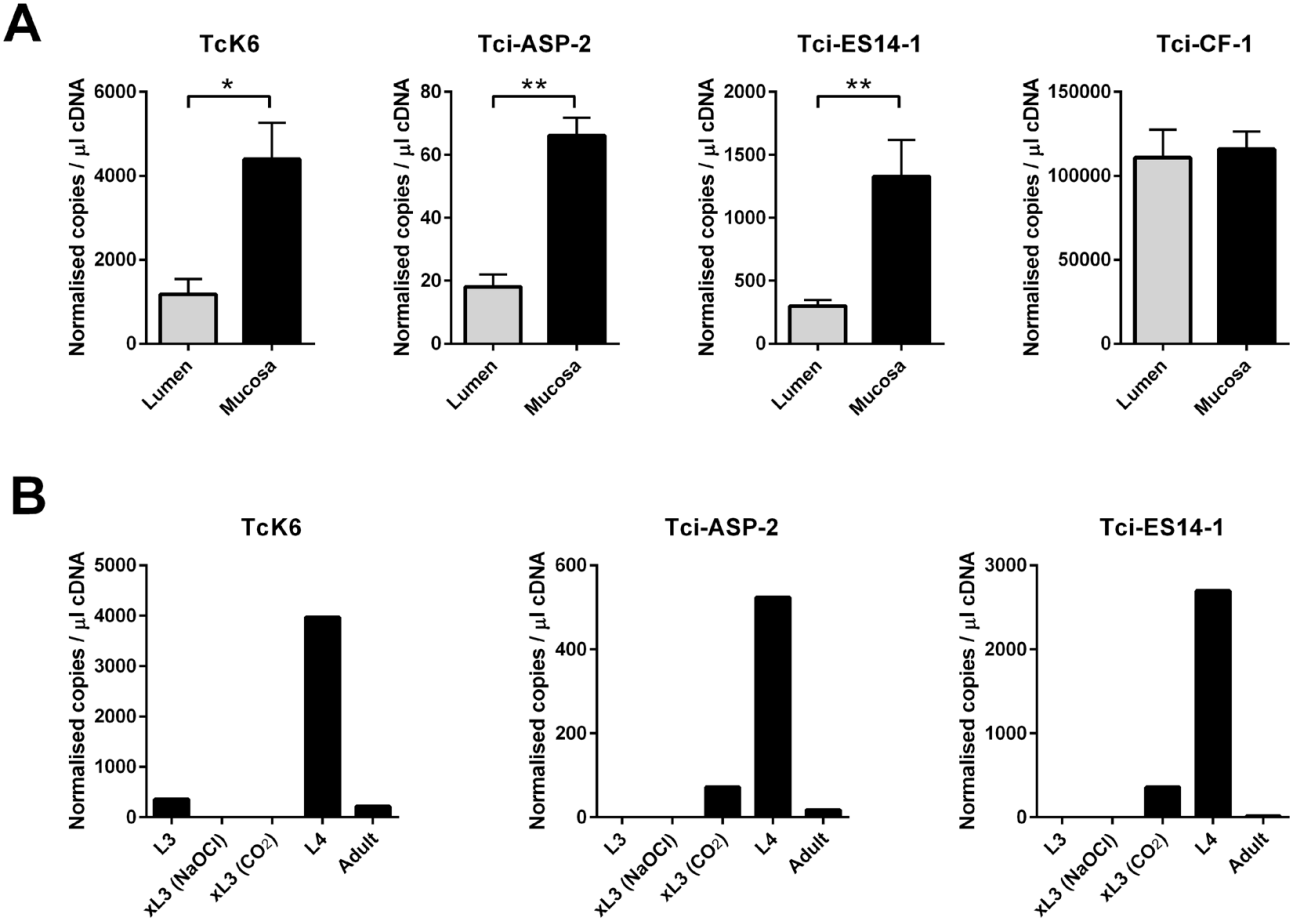
qPCR analysis of selected genes identified from RNA-Seq analysis. (**A**) qPCR validation of three differentially expressed transcripts (TcK6, Tci-ASP-2 and Tci-ES14-1) and one non-differentially expressed transcript (Tci-CF-1) identified through RNA-Seq analysis. (**B**) Stage specific transcription of TcK6, Tci-ASP-2 and Tci-ES14-1 as determined by qPCR. xL3 (NaOCl): *T. circumcincta* L3 larvae exsheathed following NaOCl treatment; xL3 (CO2): *T. circumcincta* L3 larvae exsheathed following treatment with CO_2_^56^; * = *P* < 0.05; **P < 0.001.

To validate the RNA-Seq analysis, we selected four isotigs for validation by qPCR. These represented three isotigs which were up-regulated in MD larvae: isotigs 05835, 20922 and 15536, representing Tci-ASP2, TcK6 and Tci-ES14-1, and one isotig (02529) which was homologous to Tci-CF-1 (accession no. ABA01328.1) and was not differentially expressed between MD and LD larvae. Isotigs for qPCR validation were selected to represent venom allergen/ASP-like proteins (Tci-ASP2) which are thought to play a key role in the parasitic stages of nematodes^14^, a ShK-domain containing protein (Tck6) with putative immunomodulatory activity^15^, and a representative of the novel family of transcripts (Tci-ES14-1) which were upregulated in MD larvae in this study. Levels of Tci-ASP2, TcK6 and Tci-ES14-1 transcripts were significantly upregulated in MD larvae compared to LD larvae, whereas Tci-CF-1 transcripts were similar in the two larval populations (Fig. 3A). Additional qPCR analysis was performed to determine the expression profile of the three MD up-regulated transcripts across the different lifecycle stages of *T. circumcincta*. The expression levels of all three transcripts (Tci-ASP2, TcK6 and Tci-ES14-1) were considerably higher in L4 larvae compared to L3, exsheathed L3 and adult parasites, further supporting a role for these transcripts in the mucosal stage of the parasite lifecycle (Fig. 3B).

### Sequence analysis of *T. circumcincta* ShK-like peptides

**Figure 4:**
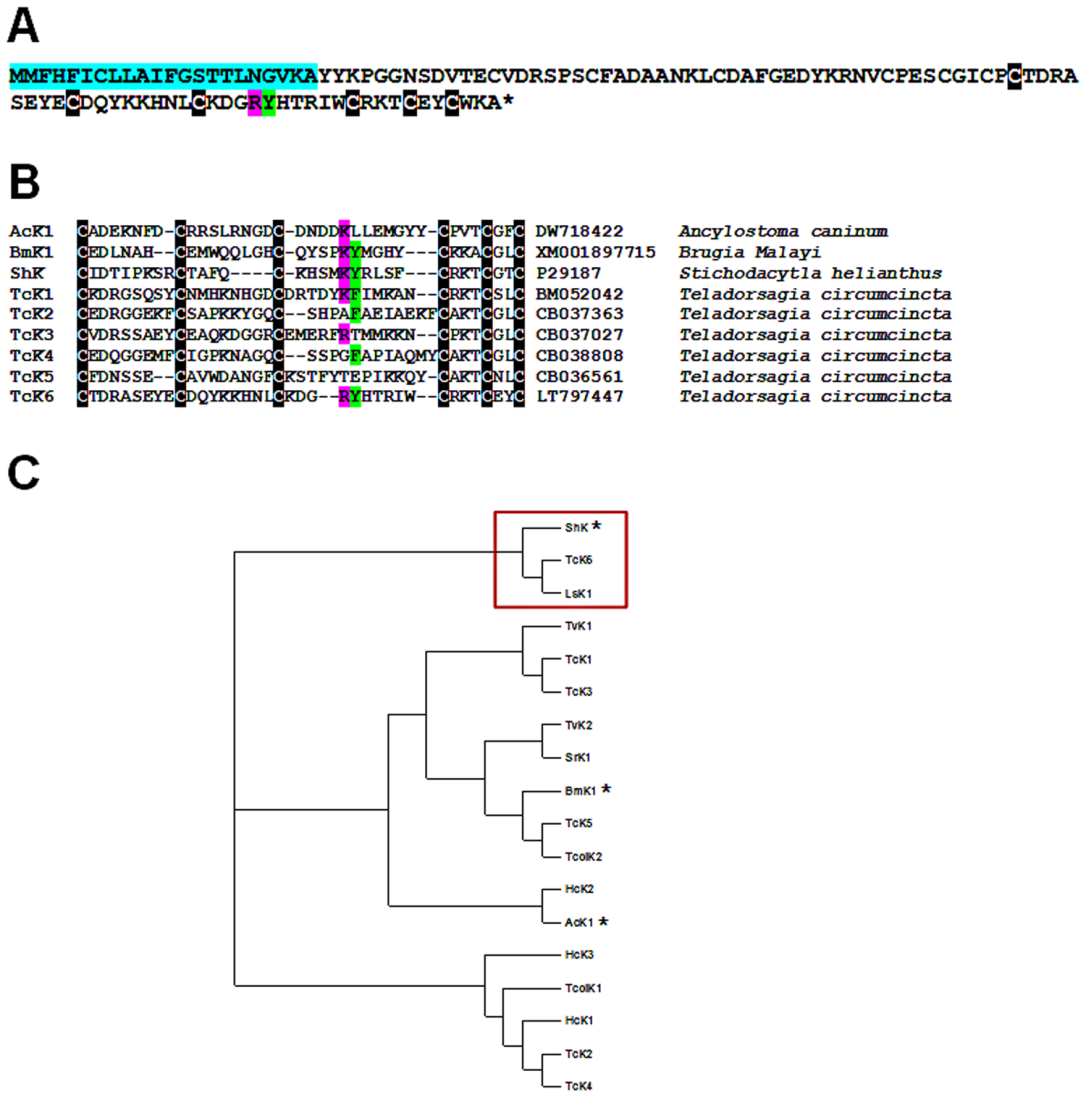
Predicted animo-acid sequence of TcK6 and relationship of TcK6 ShKT domain with those from related proteins from a variety of eukaryotic organisms. (**A**) Full-length coding sequence of TcK6. Signal peptide is indicated in blue. (**B**) Alignment of ShKT domain amino acid sequences from *Ancylostoma caninum* (AcK1), *Brugia malayi* (BmK1), *Stichodactyla helianthus* (ShK) and *Teladorsagia circumcincta* (TcK1-6). For panels (A) and (B), conserved cysteines are highlighted in black, the K/R residue which is critical for Kv1.3 channel inhibition is highlighted in magenta and the neighbouring aromatic residue (F or Y) highlighted in green. (**C**) Alignment of 35–40 amino acid residue ShKT domains derived from EST or protein depositions in public databases with TcK6 using ClustalX (version 2.0.10). A relationship tree was constructed using TreeView. *T. circumcincta*-derived molecules: TcK1 BM052042; TcK2 CB037363; TcK3 CB037027; Tck4 CB038808; TcK5 CB036561; TcK6 LT797447. *Lepeophtheirus salmonis*-derived LsK1 Protein ZK643.6 ADD24043. *Haemonchus contortus*-derived molecules: HcK1 CB012943; HcK2 AW670823; HcK3 CB012425. *A. caninum*-derived: AcK1 DW718422. *Strongyloides ratti*-derived: SrK1 CEF66800. *Brugia malayi*-derived BmK1 XM_001897715; *Stichodactyla helianthus*-derived ShK P29187; *Trichostrongylus vitrinus*-derived TvK1 AJ616691 and TvK2 AJ616650^63^; *Trichostrongylus colubriformis*-derived TcolK1 Tcol3810712 from HelmDB tcol_prot.fa; TcolK2 Tcol3698983 from HelmDB tcol_prot.fa (www.helmbd.org)^64^. The red box highlights TcK6 and the asterisks denote molecules with previously described immunomodulatory function^13^.

ShKT-domain containing proteins from parasitic nematodes have recently been shown to possess immunomodulatory activity via blockage of voltage-gated potassium (Kv) 1.3 channels on human effector memory T cells (T_EM_)^15^. As the TcK6 isotig contained a ShKT domain, this was selected for further analysis. Full length coding sequence (CDS) for Tck6 was obtained and the transcribed sequence is shown in Fig. 4A. This encoded a predicted 12.7 kDa protein with a signal peptide and a C-terminal ShKT domain. A manual alignment of the ShKT-domain of TcK6 with ShKT-domains from the archetypal ShK protein from the Caribbean sea anemone*, Stichodactyla helianthus* (ShK) and ShKT proteins from *Ancyclostoma caninum* (AcK1) and *Brugia malayi* (BmK1), all of which possess immunomodulatory activity^15^, and previously identified ShKT-containing transcripts from an adult *T. circumcincta* cDNA library (TcK1-5) is shown in Fig. 4B. Isotigs representing TcK1-5 were present in the transcriptomes of both MD and LD larvae within this study but were not differentially expressed between the two populations. All sequences contained the six conserved cysteine residues typical of the ShKT-domain. TcK1 and TcK6 also contained a positively charged residue (Arg or Lys) and an adjacent aromatic/hydrophobic reside (Phe or Tyr) between the third and fourth cysteine residues which is necessary for potent Kv1.3 channel blockade in other ShKT-like proteins^15–17^.

To further explore the relationship between TcK6 and other previously identified ShKT-domain containing proteins, the 35–40 amino acid residues forming the ShKT domains (IPR003582, PF01549) of *S. helianthus* ShK and a selection of parasitic eukaryotes were derived from EST or protein depositions in public databases and aligned with TcK6 using ClustalX (version 2.0.10). Relationships amongst the molecules were inferred by Neighbour-joining analysis and the resulting dendogram was constructed using TreeView (Fig. 4C). In agreement with the results of BLASTx analysis of the CDS of TcK6, the ShKT domain of the molecule grouped closely with those from the archetypal ShKT from *S. helianthus* and the sea louse protein ZK643.6.

### Immunomodulatory activity of Tck6

**Figure 5:**
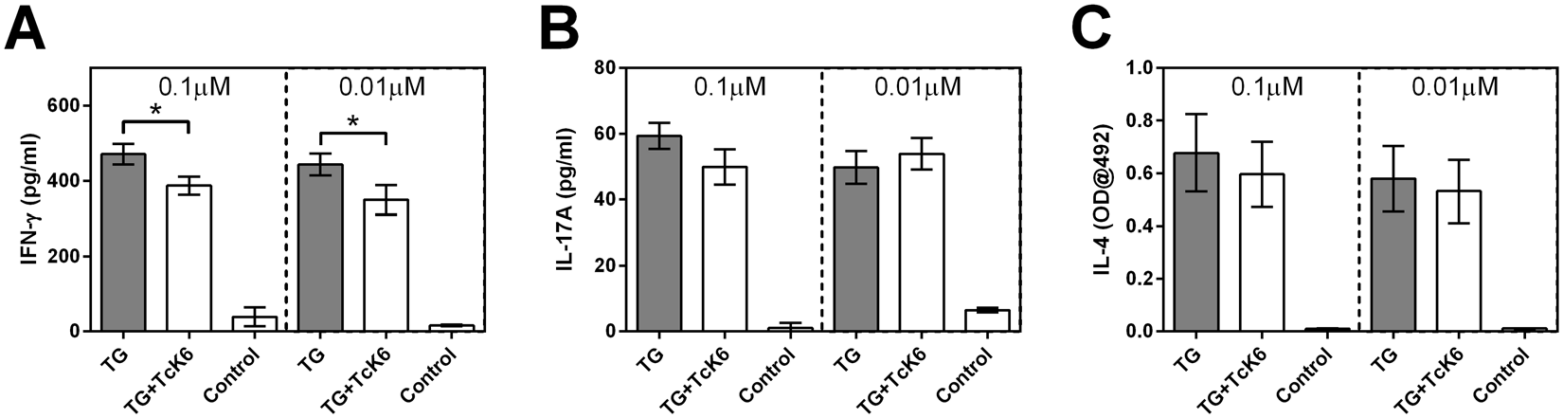
TcK6-mediated inhibition of cytokine production from activated ovine PBMC. Ovine PBMC isolated from 4 adult helminth-free sheep were stimulated in triplicate wells with thapsigargin (TG) in the presence or absence of 0.1 µM or 0.01 µM TcK6 (indicated in each graph) and the levels of (**A**) IFN-γ, (**B**) IL-17A and (**C**) IL-4 released into the culture supernatant quantified by ELISA. Addition of both concentrations of TcK6 significantly reduced the levels of IFN-γ released by TG-stimulated PBMC but had no effect on release of IL-17A or IL-4. P-values from two-tailed Mann-Whitney *t-tests* are indicated for statistically significant differences between TG stimulated cells with or without TcK6 (n = 12). Control = PBMC without TG or TcK6 stimulation. * = *P* < 0.05.

To test the immunomodulatory properties of TcK6, full length TcK6 protein without the signal peptide was synthesised and tested for the ability to modulate cytokine production by activated ovine PBMC. Thapsigargin, a SERCA pump inhibitor, was used to induce cytokine production by T cells^18, 19^. As shown in Fig. 5, nanomolar concentrations of TcK6 suppressed thapsigargin-stimulated IFN-γ production by ovine PBMC (*p* < 0.05) but had no effect on IL-17A or IL-4 production.

## Discussion

We have demonstrated here, for the first time, that the expression of a suite of genes is differentially regulated in a niche-specific manner between two populations of a single developmental stage of a parasitic nematode species within it’s host. The functions of several of the molecules that are up-regulated in mucosal-dwelling *T. circumcincta* L4 worms remain obscure beyond their identification as probable excretory/ secretory molecules which occur in expanded families. For other differentially-expressed molecules, the proposed functions are clearer.

Tck6, upregulated in MD larvae, belongs to a family of ShKT-related proteins. First described in the sea anenome *S. helianthus*, it is now apparent that ShKT-like proteins are produced by a large number of parasitic nematodes, including the economically important ruminant parasites *T. circumcincta* ^15^, *Haemonchus contortus* ^15^, *Trichostrongylus colubriformis* ^20^ and *Ostertagia ostertagi* ^3^, where they can contain single or multiple ShKT domains. These proteins are commonly found in nematode secretomes^21–23^ and can be upregulated during parasitic stages of the nematode life-cycle^3^, suggesting a role in the host:parasite interaction. Recently a role for ShKT-like proteins in parasite-mediated immune modulation has been described^15^. This immunomodulatory activity is related to ShK-toxin mediated blockage of the voltage-gated potassium (Kv) 1.3 channel which plays a critical role in activation of T_EM_ cells by regulating membrane potential and Ca^2+^ signaling during T cell activation^18^. Other T cell subsets are refractory to Kv1.3 blockade as they use a different potassium channel, namely the calcium-activated potassium channel KCa3.1, during the activation process^24^. T_EM_ cells are thought to confer immediate protection following re-exposure to pathogens by surveying mucosal barriers or diseased tissues and displaying immediate effector function upon recognition of their cognate antigens^25, 26^. Thus the targeting of T_EM_ by the parasitic stages most closely associated with the mucosa would appear to be a useful strategy for immune evasion by the parasite.

In this study we have shown that TcK6 contains key residues within its single ShKT-domain predictive of functional Kv1.3 channel blockade, shared a high sequence similarity to the archetypal ShKT protein ShK from *S. helianthus*, and was capable of suppressing IFN-γ production by thapsigargin-activated ovine T cells in a similar manner to that seen with the parasite-derived ShKT proteins, AcK1 (from the hookworms *Ancyclostoma caninum* and *A. celynum*) and BmK1 (from *Brugia malayi*). The lack of suppression of IL-17A and IL-4 production by TcK6 is consistent with previous studies which found that *S. helianthus* ShK was more effective at suppressing production of T_H_1-associated compared to T_H_2 or T_H_17 cytokines^18, 27^. As thapsigargin results in T cell activation without upregulating KCa3.1 channels^18^, the assays employed in this study did not specifically interrogate the effects of TcK6 on T_EM_ cells, as other T cell subsets within the PBMC population would be equally susceptible to the effects of Kv1.3 blockade. Nevertheless, these results suggest that TcK6 may play a role in immune evasion by *T. circumcincta* L4, particularly as a prominent role for mucosal T cells in immunity against this parasite has been described^28^. The additional observation that TcK6 transcripts are significantly upregulated in L4 compared to L3 and adult stage *T. circumcincta*, which are less closely associated with the abomasal mucosa, provides further evidence for a role of Tck6 in modulating the local mucosal environment.

Several of the transcripts identified as up-regulated in the mucosal-dwelling larvae were classified as activation-associated secretory proteins (ASPs). These nematode-specific proteins are members of the SCP/Tpx-1/Ag5/PR-1/Sc7 family, a diverse, evolutionarily-related, group of secreted proteins identified in a wide range of organisms^14^. ASPs are abundant in the transcriptomes and ES material of L4 *T. circumcincta* ^6, 29^ and, although their function(s) are likely to be diverse, it has been suggested that they are key to the transition of nematodes from the free-living to the parasitic state^30, 31^, having a role in maintenance and/or exit from arrested development^32^, in modulation of the host immune response^33^ and in maintenance of the parasites within their niche in the host^34, 35^. Recently a specific immunomodulatory function was demonstrated for Na-ASP-2 from the human hookworm *Necator americanus*: this ASP bound CD79A, a component of the B-cell antigen receptor complex, on protein microarrays and specifically bound *ex-vivo* human B cells^36^. In the latter, the binding of Na-ASP-2 led to the down-regulation of nearly 1000 transcripts in human B cells, including critical components of B cell receptor signaling pathways^36^. Although homology-searching using Blastp identified Tci-ES14 as a homologue of a putative L3 ES protein of unknown function from *O. ostertagi*, structural and functional prediction using I-TASSER^37^ demonstrated the highest structural match for Tci-ES14 to be Na-ASP-1 (TM score 0.715), further demonstrating the importance of SCP/Tpx-1/Ag5/PR-1/Sc7 family and similar proteins in host parasite interaction for *T. circumcincta*.

Nematode ASPs have previously shown great promise as vaccine candidates against parasitic hookworms in humans, though induction of generalized urticaria following immunisation of pre-exposed individuals has now halted the development of a vaccine based on these molecules^38^. From a veterinary perspective, immunisation of sheep with a preparation from *H. contortus* enriched for a C-type, single-domain ASP, “Hc24”, as well as a non-ASP 15 kDa protein, gave reductions of over 70% in both mean worm burdens and faecal egg counts^39, 40^. The N-type single domain ASPs, Oo-ASP-1 and Oo-ASP-2, are the principal components of an ASP-enriched native extract of adult *O. ostertagi* which has been used with success in vaccination trials in cattle^41–43^ and, in *T. circumcincta*, a homologue of Oo-ASP-1 is a component of a recombinant vaccine cocktail which has convincingly provided protection in repeated vaccine trials in sheep^44^.

Studies in which sheep have been infected with bolus doses or trickle-infections of *T. circumcincta* and subsequently euthanased at different time-points have shown that some worms are intimately associated with the mucosa whereas others are present in the lumen (e.g. refs 7, 9 and 44). These studies show only snapshots of what may be a more complex scenario: It may be the case that worms do not necessarily populate one niche or the other but in fact move between the two niches regularly, turning on the expression of suites of mucosal-associated genes at the appropriate time and then downregulating these transcripts when they re-emerge into the lumen: the observation that both mucosal and luminal dwelling larvae in this study were of the same developmental stage would support this hypothesis. Such movement of worms between different niches may also be true for other parasitic nematodes which share a luminal and tissue dwelling stages and warrants further investigation.

The niche-specific transcriptomic analysis described in this study allows the investigation of a relatively small number of transcripts which may be of very high value in answering basic biological questions about host-parasite interactions. For many parasitic nematodes it is not possible to answer these basic biological questions by using functional tools, such as genome-wide RNA interference studies, that are employed in many other eukaryote systems^45^. However, the reductionist approach of studying niche-specific transcripts, demonstrated here distils the list of molecules for functional analysis such that more expensive, but potentially effective, methodologies such as the use of phosphorodiamidate morpholino oligomers (PPMOs)^46^, which demonstrate gene function by translational silencing of the gene products, becomes realistic. The assignation of gene function becomes even more challenging when either no homology exists to molecules in public databases (as discussed in ref. 47) (which was the case for 54% of the transcripts with enriched expression in MD larvae here) or where no function has been ascribed to the homologue. For both of these categories of molecule (no homology/unknown function) it is important to start to build a profile to understand gene function; niche specific expression is a very good starting point and can then be augmented with technologies to demonstrate tissue specificity (single cell transcriptomics, immunolocalisation, *in situ* hybridisation) and gene function analysis (e.g. PPMO) to illuminate gene function in host:parasite interactions and thus potential for rational intervention.

In conclusion, this study has identified a subset of niche- rather than developmental stage-specific transcripts of *T. circumcincta* larvae. Most transcripts were up-regulated in mucosal- compared to lumen-dwelling larvae, suggesting that they encode for proteins which are important for survival of the parasite within the mucosal environment. In support of this, one of the upregulated transcripts encoded for a novel immunomodulatory protein, TcK6, which may be involved in regulating local T cell memory responses. Niche-specific analysis of parasites represents a promising approach to identify genes and molecules essential for within-host survival, particularly where parasites of identical or similar developmental stages exist in different host niches.

## Methods

### Ethics statement

All experimental procedures described here were approved by the Moredun Research Institute Experiments and Ethics Committee and were conducted under the legislation of a UK Home Office License (reference PPL 60/03899) in accordance with the Animals (Scientific Procedures) Act of 1986.

### Experimental *T. circumcincta* challenge studies and isolation of *T. circumcincta* fourth stage larvae (L4)

Six Texel cross lambs of three months of age reared under conditions to preclude accidental exposure to helminth parasites were challenged per os with 150,000 infective *T. circumcincta* larvae (isolate MTci-2_CVL). All of the animals were necropsied seven days post-challenge; the methods used to euthanase the animals and remove their organs for worm burden recovery were as described previously^48^. The abomasum was split along the greater curvature, contents emptied into a labeled container, and washed in physiological saline (PS; 0.85% NaCl w/v). For each individual animal, the abomasal contents and washings were combined together and processed as described below, to generate a ‘lumen dwelling (LD)’ population of worms: In brief, the combined contents and washings were allowed to settle and the supernatant discarded; the resultant digesta was encapsulated within a double layer of surgical muslin, suspended in PS and incubated at 37 °C for four hours. The worms migrating through the muslin were collected from the PS and this constituted the LD worms from each animal.

The ‘mucosal dwelling (MD)’ population was generated by pinning out the washed abomasum on to a polystyrene board, suspending it in PS and incubated at 37 °C for four hours. The migrating worms were collected from the PS.

### Parasitological measurements

Fifty individual juvenile worms were randomly selected from each lamb within a group, mounted in 2% formalin (v/v) on a microscope slide, examined for sex, stage of development assessment^8^, and photographed (Nikon D70), at magnification (x40) on a compound microscope to enable body length to be determined. Where multiple images were captured, the files were compiled to form a single composite using an image editing program (Adobe Photoshop Elements 8.0). Worm lengths were estimated using image processing software (ImageJ 1.46r).

### Generation of the *T. circumcincta* transcriptome

In order to generate the *T. circumcincta* transcriptomic database, raw read data was obtained from the following resources as described in ref. 49 and consisted of a total of 1,188,329 sequence reads from five independent in-house databases (detailed in Table 3) including: (A) *T. circumcincta* EST database generated by suppressive subtractive hybridisation (SSH)^6^; (B) *T. circumcincta* L4 larvae EST database generated by 454 next generation sequencing (NGS) (Unpublished); (C) *T. circumcincta* L3 larvae EST database generated by 454 NGS^50^; (D&E) *T. circumcincta* adult EST databases generated by 454 NGS^51, 52^. Assembly of raw reads was performed using Newbler 2.9 (Roche 454) sequence analysis software. The level of completeness of the *T. circumcincta* transcriptome assembly was assessed via Benchmarking Universal Single-Copy Orthologs (BUSCO) analysis^13^ using the Nematoda lineage dataset as a reference. The *T. circumcincta* transcriptomic database used for this study is freely available via the Moredun Research Institute website (http://www.moredun.org.uk/research/teladorsagia-circumcincta-transcriptome) or via the corresponding author on request.

**Table 3:** Summary of in-house databases used to construct the *T. circumcincta* transcriptomic database.

Database	Accession No.	Sequences	Reference
A	AM743198–AM744942	1,768	6
B	N/A	507,124	N/A
C	ERR039830-ERR039823	168,131	50
D	ERR016356-ERR016357	99,710	51
E	SRR328404 and SRR328405	411,596	52

### Illumina Solexa RNA-seq (Hi-Seq) analysis of niche-specific gene expression in *T. circumcincta* L4

RNA was extracted from three separate samples (biological replicates) of luminal or mucosal isolated *T. circumcincta* larvae using Trizol reagent (Life Technologies, UK) using a using a pestle and mortar on dry ice. RNA quality was assessed on a spectrophotometer (Nanodrop, Thermo Scientific Ltd, UK) with OD260/280 ranging from 1.89–2.1. Samples of sufficient quality for subsequent analysis were obtained from three paired MD and LD populations from three individual sheep. RNA (5 µg of each sample) was processed by Edinburgh Genomics sequencing service, University of Edinburgh for Illumina Solexa Hi-Seq sequencing following the manufacturer’s protocol^53^. Base calls were made using the Illumina CASAVA 1.8 pipeline. Post-sequencing, read quality of raw FASTQ files was checked with FastQC v0.10.1^54^. The CLC Genomics Workbench (Version 8, Qiagen Ltd) was then used for adapter, quality, ambiguity, and length trimming; alignment of the RNA-seq data with the *T*. c*ircumcincta* transcriptome and generation and normalisation of the reads *per* kilobase *per* million mapped reads^55^. Allowing the total number of reads for each of the six RNA sample sets, which mapped to each contig/isotig of the *T. circumcincta* transcriptome to be determined.

### Quantitative PCR (qPCR) of *T. circumcincta* transcript expression

Oligonucleotide primers were designed to amplify regions of 169–474 bp, dependent on transcript, for each of *Tci-asp-2*, *Tci-es14*, *Tck6, Tci-tub-1* (β tubulin, accession number Z69258.1) and *Tci-cf-1* (Cathepsin F, accession number DQ133568.1). The sequences for these primers are available from the authors on request. Using 0.8 μg of total RNA extracted from L4 *T. circumcincta* derived from the mucosal subset recovered from lamb number 1369 as a template, reverse transcription PCR (RT-PCR) was performed to amplify cDNA representing each of the transcripts. The SuperScript® III One-Step RT-PCR System with Platinum® *Taq* DNA Polymerase (Invitrogen) was employed, following manufacturer’s instructions. PCR-amplified cDNA representing each of the transcripts was ligated into the pGEM^®^-T Easy vector (Promega) and transformed by heat shock into competent *Escherichia coli* (strain JM109, Promega). Plasmids derived from cultures of these transformed cells were sequenced (eurofins genomics) and used as standards in qPCR employing nested gene-specific primers.

cDNA was generated from RNA samples used for previous Illumina Solexa Hi-Seq sequencing, plus an additional 3 samples from MD larvae and 1 sample from LD larvae (representing a total of 6 MD and 4 LD populations), using SuperScript® II Reverse Transcriptase (Invitrogen) and oligo(dT)_23_ primer (Sigma) according to manufacturer’s instructions. Real-time PCR amplification was performed using ABI PRISM® 7000 Sequence Detection System, and SYBR® GreenER™ qPCR SuperMix (Invitrogen), in 25 μl volumes using 1 μl cDNA and 0.2 μM of each primer. For all primer sets detailed, cycling conditions were as follows; 50 °C for 2 mins, 95 °C for 10 mins, 40 cycles of 95 °C for 30 s, 55 °C for 30 s and 72 °C for 30 s. Fluorescence was detected at the end of each cycle and following completion of 40 cycles, product dissociation performed by one cycle of 95 °C for 15 s, 60 °C for 20 s and 95 °C for 15 s. For each gene analysed, a standard curve was performed using appropriate plasmids, previously constructed and detailed earlier, from 10^8^ to 10^1^ copies per μl of plasmid. The number of copies per μl of cDNA was then normalized using results for β-tubulin (*Tci-tub-1*), as a housekeeping gene.

Validation of Illumina Solexa Hi-Seq sequencing was performed using *Tci-cf-1*, 05838, 15536 and 20922 genes. These genes were also assessed for stage specificity using cDNA (kindly supplied by Thomas Tzelos, Moredun Research Institute) which were from L3, exsheathed L3 (NaOCl), exsheathed L3 (CO_2_), L4 and Adult cDNA samples^56^.

### Synthesis of Tck6

Full length coding sequence (CDS) for Tck6 was determined using a PCR amplification strategy: Gene specific primers for Tck6 were designed, based on the available contig sequence for this transcript, to amplify the 5′ and 3′ termini of cDNA representing the transcript when used in conjunction with vector-specific primers and employing a cDNA library [constructed using *T. circumcincta* L4 (8 days post infection, dpi) RNA in λTriplEx2] as template^57^ and Platinum® *Taq* DNA Polymerase (Invitrogen). Analysis of the resulting CDS showed the presence of a putative signal peptide (predicted using SignalP 4.0^58^, which was omitted during the synthesis of the remaining polypeptide (Activotec Ltd, UK).

### Cytokine production by Tck6 stimulated ovine peripheral blood mononuclear cells (PBMC)

PBMC were isolated from four adult helminth-free Texel cross sheep using a Ficoll-Paque™ PLUS density gradient (GE Healthcare, Little Chalfont, UK) as previously described^59^ and assays performed in triplicate for each source animal. PBMC were cultured in 96-well round-bottomed plates at 5 × 10^5^ cells/well in RPMI 1640 (Invitrogen) containing 10% foetal calf serum (FCS), 2 mM L-glutamine, 100 U/mL penicillin, 100 μg/mL streptomycin and 50 μM 2-mercaptoethanol at 37 °C 5% CO_2_. Cells were pre-incubated for 18 h with 0.1 µM Tck6 polypeptide, 0.01 µM Tck6 polypeptide, or media alone prior to stimulation with 0.4 µM Thapsigargin (Sigma-Aldrich) for 48 h. Cells which had not been incubated with Tck6 or Thapsigargin acted as unstimulated controls. As both TcK6 and Thapsigargin were initially solubilised in DMSO before dilution in culture medium, an equivalent concentration of DMSO in culture medium was added to the relevant unstimulated control wells. Cell-free culture supernatants were harvested and stored at −70 °C prior to analysis. Levels of IFN-γ, interleukin (IL)-4 and IL-17A within the supernatants were quantified using a commercial ELISA kit according to the manufacturer’s instructions (MABTECH AB, Augustendalsvägen, SE, Sweden for IFN-γ and IL-4; Kingfisher Biotech for IL-17A) and expressed as pg/mL.

### Statistical analyses

The statistical package DESeq. 2 (Version 1.8.1), within the R software suite (Version 3.1)^60^ was used to analyse the RNA-seq (Illumina Hi-Seq) data and to identify transcripts significantly differentially expressed between LD and MD *T. circumcincta* larvae using a model based on a negative binomial distribution on variance estimated and size factor normalized data taking into account the 3 biological replicates for each of the two conditions^61^. Read count data for each replicate of each condition were normalised using RPKM (reads per kilobase per million mapped reads) as described previously in ref. 55. A minimum RPKM value of ≥1 was required for further analysis. Significantly differentially expressed transcripts were classified as those having a fold change ≥±1.5 between luminal and mucosal larvae samples and a False Discovery Rate (FDR) corrected p-value of ≤0.05^62^. Putative functions were assigned to the differentially expressed transcripts following homology searches using the NCBI nr database (25^th^ July 2014 release) and motif identification using IPS within the Blast2GO pipeline. In addition, differentially expressed transcripts were mapped onto the *T. circumcincta* genome assembly (PRJNA72569): http://parasite.wormbase.org/Teladorsagia_circumcincta_prjna72569. The NGS data from this study is fully compliant with the MINSEQE guidelines and has been deposited in the publically available NCBI SRA Database under the following accession number: SRP096620.

Parasitological and cytokine-release data was analysed using GraphPad Prism version 6.05 for Windows (GraphPad Software, La Jolla California USA, www.graphpad.com). Worm length data (which was normally distributed) was analysed using 2-way ANOVA with site and sex as factors. Larval male:female ratios in MD and LD populations (also normally distributed) were analysed using a students *t-test*. Stage of larval development, qPCR data and cytokine release data, which were not normally distributed, were analysed using a two-tailed Mann-Whitney *U* *-test*. *P* values of <0.05 were considered significant.

## Electronic supplementary material

Supplementary Information
