# Supplementary material for: Niche-specific gene expression in a parasitic nematode; increased expression of immunomodulators in Teladorsagia circumcincta larvae derived from host mucosa
Source: Sci Rep. 2017 Aug 3;7:7214. doi: 10.1038/s41598-017-07092-0 (PMC5543109; doi:10.1038/s41598-017-07092-0)

Niche-specific gene expression in a parasitic nematode; increased expression of immunomodulators in *Teladorsagia circumcincta* larvae derived from host mucosa

Tom N McNeilly[1]*, David Frew[1], Stewart TG Burgess[1], Harry Wright[1], David J. Bartley[1], Yvonne Bartley[1], Alasdair J. Nisbet[1]*

[1]Moredun Research Institute, Edinburgh, United Kingdom

* Corresponding authors:
Tom.McNeilly@moredun.ac.uk

Alasdair.Nisbet@moredun.ac.uk

Supplementary Information

Table S1: Details of isotigs differentially expressed in mucosal vs. luminal *T. circumcincta* L4 with no BLAST hits, with FDR adjusted p-value ≤0.05.

Isotig accession no.	Tci-gene ID*	Fold change	Signal Peptide
isotig14360	TELCIR_04003	9.55	No
isotig13538	No hits found	6.37	Yes
isotig14454	TELCIR_02385	4.37	Yes
isotig18429	TELCIR_13609	3.95	Yes
isotig22967	TELCIR_00024	3.72	Yes
isotig16824	TELCIR_04250	3.68	Yes
isotig06111	No hits found	3.32	Yes
isotig16701	No hits found	3.23	Yes
isotig25735	No hits found	2.95	No
isotig16523	No hits found	2.68	Yes
isotig19933	TELCIR_17994	2.63	Yes
isotig16072	No hits found	2.53	Yes
isotig17141	No hits found	2.45	No
isotig22158	TELCIR_13609	2.37	Yes
isotig16367	TELCIR_13609	2.25	Yes
isotig15198	No hits found	2.11	Yes
isotig13966	No hits found	2.09	No
isotig13903	TELCIR_02385	1.96	Yes
isotig17221	TELCIR_15090	1.93	Yes
isotig25639	TELCIR_17448	1.90	Yes
isotig18187	No hits found	1.90	No
isotig18220	No hits found	1.89	Yes
isotig15135	TELCIR_00431	1.87	Yes
isotig19257	TELCIR_13609	1.79	Yes
isotig17976	TELCIR_15090	1.70	Yes
isotig19940	TELCIR_04250	1.69	Yes
isotig06999	No hits found	1.60	No
isotig15284	No hits found	1.59	No
isotig17985	No hits found	1.57	Yes
isotig23208	TELCIR_13609	1.51	Yes
isotig18078	TELCIR_00024	1.50	No

*identity of homologous gene from *Teladorsagia circumcincta* genome assembly: (PRJNA72569): http://parasite.wormbase.org/Teladorsagia_circumcincta_prjna72569

Supplementary Figure 1. Functional grouping of proteins encoded by each of the differentially-expressed molecules identified in lumen-dwelling and mucosal-dwelling L4 larvae of *Teladorsagia circumcincta*. Relationships were inferred by alignment of nucleotide sequences using Clustalx version 2.0.10, employing Neighbour-joining analysis and the resulting relationship tree was constructed using TreeView. Function was ascribed based on homology-searching of the NCBI nr database using BLASTx.

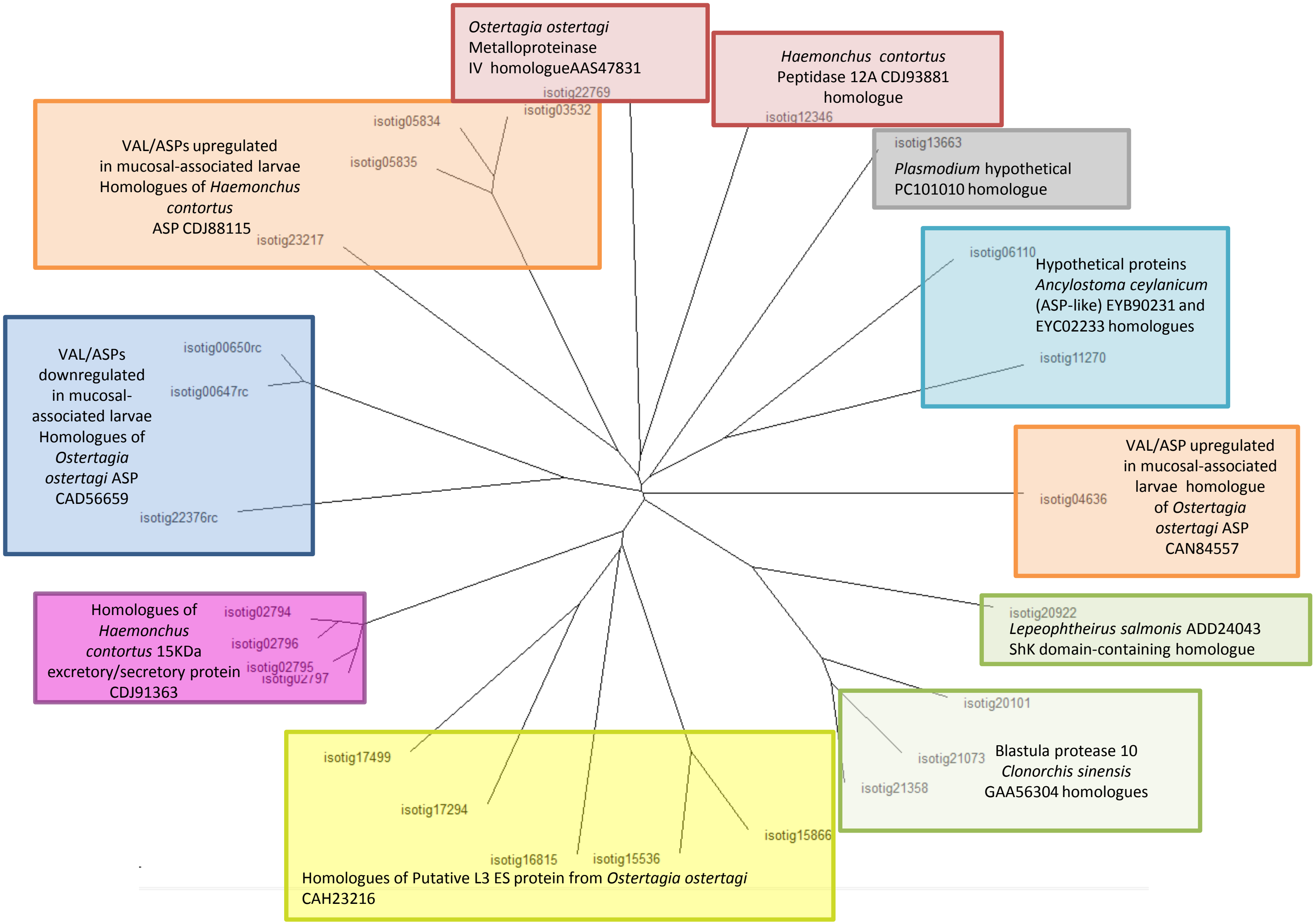

Supplement: Supplementary file 1 — Supplementary Information [file 41598_2017_7092_MOESM1_ESM.pdf]
